# Association Between Chemotherapy and Survival in T1 Colon Cancer With Lymph Node Metastasis: A Propensity-Score Matched Analysis

**DOI:** 10.3389/fonc.2021.699400

**Published:** 2021-07-30

**Authors:** Wangxin Yan, Huizhen Zhou, Si Shi, Jixu Lin, Qiangkang Lin

**Affiliations:** Department of Colorectal and Anal Surgery, The Third Affiliated Hospital of Shanghai University, Wenzhou People’s Hospital, Wenzhou No. 3 Clinical Institute Affiliated to Wenzhou Medical University, Wenzhou, China

**Keywords:** chemotherapy, survival, T1, colon cancer, propensity score

## Abstract

This study aimed to comprehensively examine the efficacy of chemotherapy in T1 colon cancer patients with lymph node metastasis.

**Methods:**

The differences in categorical variables in colon cancer patients according to lymph node status were evaluated by Pearson’s chi-square test. The Kaplan-Meier method was used to assess Cancer-specific survival (CSS) and overall survival (OS) with the log-rank test. Cox proportional hazards models were built, multivariate Cox regression analyses were performed with the hazard ratio (HR) and 95% confidence interval (CI) to identify the potential independent prognostic factors. Propensity score matching was also undertaken to adjust for treatment bias due to measured confounders.

**Results:**

Younger age (52.2% VS. 43.0% for ≤ 65 years old, p < 0.001), female gender (50.3% VS. 46.8% for female, p < 0.001), more lymph nodes harvested (68.1% VS. 46.6% for ≥12 lymph nodes harvested, p < 0.001), Black race (13.6% VS. 12.0% for the Black race, p < 0.001), and higher tumor grade (14.2% VS. 5.6% for grade III/IV, p < 0.001) were more prone to be diagnosed with lymph node involvement. The receipt of adjuvant chemotherapy following radical surgery significantly reduced the risk of colon cancer-specific mortality by 33.9% after propensity-score matching (HR = 0.661, 95%CI = 0.476-0.917, p = 0.013).

**Conclusions:**

Younger age, female gender, more lymph nodes harvested, Black race, and higher tumor grade were more prone to be diagnosed with lymph node involvement. The receipt of adjuvant chemotherapy following radical surgery also significantly decreased the risk of colon cancer-specific mortality by 33.9% in T1 colon cancer with lymph node involvement.

## Introduction

Colon cancer is among the most common causes of cancer and cancer-related death (1). T1 colon cancer refers to carcinoma with invasion confined to the submucosa (2, 3). As reported, however, approximately 10% of T1 colon cancer patients experience lymph node metastases and require radical intestinal resection with lymph node dissection (4). Although the risk factors for lymph node metastasis in T1 colon cancer have been widely reported, differences in opinion do exist (5, 6).

The oncological outcomes of stage I colon cancer patients are generally excellent following curative surgery; however, the presence of lymph node metastasis represents a prognostic feature in poor prognosis. Among treatments, 5‐FU‐based chemotherapy has been demonstrated to have significant survival benefits for patients with lymph node metastasis (7–9). Despite this, some patients do not receive further chemotherapy following radical surgery (10). The available data of oncological outcomes in T1 node-positive (N+) patients is lacking. For example, the well-known MOSAIC study, assessing the impact of adjuvant chemotherapy for stage III colon cancer, did not include T1 disease (9).

T1 disease is relatively rare and represents a small proportion of cases of colon cancer. It has been reported that such patients account for 2 to 12 percent of all cases of colon cancer in colonoscopic studies (11–15). Therefore, a large population-based cohort is needed to evaluate the predictors for lymph node metastasis in T1 colon cancer following curative surgery. The present study aimed to comprehensively examine the efficacy of chemotherapy in T1 colon cancer patients with postoperative lymph node metastasis.

## Material and Methods

Sponsored by the National Cancer Institute (NCI), the Surveillance, Epidemiology, and End Results (SEER) database included both the incidence, clinicopathological information, and survival characteristics of malignant tumors, and covered 28% of the US population from 18 established cancer registries across the USA. The SEER*Stat software, version 8.3.8 (Surveillance Research Program, National Cancer Institute) was utilized to acquire data for this population-based study from the SEER database. Because data from the SEER database were anonymous and publicly available, ethical approval was waived and informed consent was unnecessary in this study.

The baseline covariates included the year of diagnosis, tumor location, age at diagnosis, the number of lymph nodes harvested, race, gender, grade, and chemotherapy based on the postcode of patients. As seen in **Figure S1**, we identified patients diagnosed with colon cancer between 2004 and 2015. Patients who met the following criteria were excluded: ① patient race was unknown, ② no positive histological confirmation, ③ non-adenocarcinoma histologies, ④ lack of active follow-up, and ⑤ without radical surgery. Finally, the targeted population was patients diagnosed with stage T1NanyM0 colon cancer, who were included in our analyses. Further analysis was conducted in stage T1N+M0 colon cancer patients. 

### Statistical Analysis

Patients’ demographic and clinical characteristics were included as follows: year of diagnosis (2004-2007, 2008-2011 and 2012-2015), tumor location [right-sided colon (from caecum to transverse colon) and left-sided colon (from splenic flexure to rectosigmoid junction)], age at diagnosis (≤65 years old and >65 years old), the number of lymph nodes harvested (≤11 and ≥12), race (white, Black and other), gender (male and female), grade (I/II, III/IV and unknown), and chemotherapy (no chemotherapy and chemotherapy).

Cancer-specific survival (CSS) and overall survival (OS) served as the endpoints. The differences of the categorical variables in colon cancer patients according to the lymph node status were analyzed by Pearson’s chi-square test. The Kaplan-Meier method was used to assess the survival with the log-rank test. Cox proportional hazards models were built and multivariate Cox regression analyses were performed with hazard ratio (HR) and 95% confidence interval (CI) to identify the potential independent prognostic factors from the variables examined, with P value less than 0.20 in univariate analyses.

Patient demographic and clinicopathological features were not balanced due to the inherent deficits of the retrospective cohort. Propensity score matching, a statistical normalization method for analyzing observational data by estimating the effects of a large number of factors that could affect treatment allocation, were then generated to balance covariates in different groups and reduce selection bias due to confounding variables (16). To provide a more robust assessment of survival outcomes, propensity score matching was performed between stage T1N+M0 colon cancer patients with and without the receipt of chemotherapy using a 1:1 nearest neighbor matching algorithm. The following variables were used to calculate propensity to receive chemotherapy: year of diagnosis, tumor location, age at diagnosis, the number of lymph nodes retrieved, patient race, gender, and tumor grade. Statistically significant levels were two-tailed and set at a P value of less than 0.05. Statistical analyses were conducted using the IBM Statistical Package for the Social Sciences (SPSS) version 23.0 software package for Windows (SPSS, Inc., Chicago, IL, USA).

## Results

### Patient Characteristics

In total, 36595 eligible colon cancer patients met the inclusion criteria, of which 33633 (91.9%) patients were diagnosed with lymph node-negativity and 2962 (8.1%) patients were diagnosed with lymph node positivity; 12428 (34.0%) patients were diagnosed between 2004 to 2007, 12496 (34.1%) patients were diagnosed between 2008 to 2011 and 11671 (31.9%) patients were diagnosed between 2012 to 2015; 17379 (47.5%) patients were right-sided colon cancer and 19216 (52.5%) patients were left-sided colon cancer; 15999 (43.7%) patients were less than 65 years old and 20596 (56.3%) patients were over 65 years old; 18891 (51.6%) patients had less than 12 lymph nodes retrieved and 17704 (48.4%) patients had more than 12 lymph nodes retrieved; 29051 (79.4%) patients were of white race, 4437 (12.1%) patients were of Black race and 3107 (8.5%) patients were other races; 19371 (52.9%) patients were male and 17224 (47.1%) patients were female; 29071 (79.4%) patients were diagnosed with grade I/II and 2320 (6.3%) patients were diagnosed with grade III/IV; 2414 (6.6%) patients received chemotherapy and 34181 (93.4%) patients did not. The demographic and clinical characteristics among the whole cohort are shown in **Table 1**. Our study found that those who were younger in age (52.2% VS. 43.0% for ≤ 65 years old, p < 0.001), had female gender (50.3% VS. 46.8% for female, p < 0.001), more lymph nodes harvested (68.1% VS. 46.6% for ≥12 lymph nodes harvested, p < 0.001), were of Black race (13.6% VS. 12.0% for Black race, p < 0.001), and who had a higher tumor grade (14.2% VS. 5.6% for grade III/IV, p < 0.001) were more prone to be diagnosed with lymph node involvement.

**Table 1:** Clinicopathologic characteristics of T1 colon cancer patients according to the lymph node status.

Variables	N0 (N=33,633)	N+ (N=2,962)	*P value*
**Year of diagnosis**			0.063
** 2004-2007**	11,480 (34.1%)	948 (32.0%)	
** 2008-2011**	11,459 (34.1%)	1,037 (35.0%)	
** 2012-2015**	10,694 (31.8%)	977 (33.0%)	
**Tumor location**			0.344
**Right-sided colon**	15,997 (47.6%)	1,382 (46.7%)	
** Left-sided colon**	17,636 (52.4%)	1,580 (53.3%)	
**Age at diagnosis (years)**			<0.001
** ≤65**	14,453 (43.0%)	1,546 (52.2%)	
**>65**	19,180 (57.0%)	1,416 (47.8%)	
**Number of lymph nodes harvested**			<0.001
**≤11**	17,946 (53.4%)	945 (31.9%)	
**≥12**	15,687 (46.6%)	2,017 (68.1%)	
**Race**			<0.001
**White**	26,799 (79.7%)	2,252 (76.0%)	
**Black**	4,033 (12.0%)	404 (13.6%)	
** Other**	2,801 (8.3%)	306 (10.3%)	
**Gender**			<0.001
** Male**	17,898 (53.2%)	1,473 (49.7%)	
** Female**	15,735 (46.8%)	1,489 (50.3%)	
**Grade**			<0.001
**I/II**	26,754 (79.5%)	2,317 (78.2%)	
**III/IV**	1,898 (5.6%)	422 (14.2%)	
**Unknown**	4,981 (14.8%)	223 (7.5%)	
**Chemotherapy**			<0.001
**No chemo**	33,077 (98.3%)	1,104 (37.3%)	
** Chemo**	556 (1.7%)	1,858 (62.7%)	

### The Efficacy of Chemotherapy in T1N+ Colon Cancer Patients Before Propensity Score Matching

**Figure 1:**
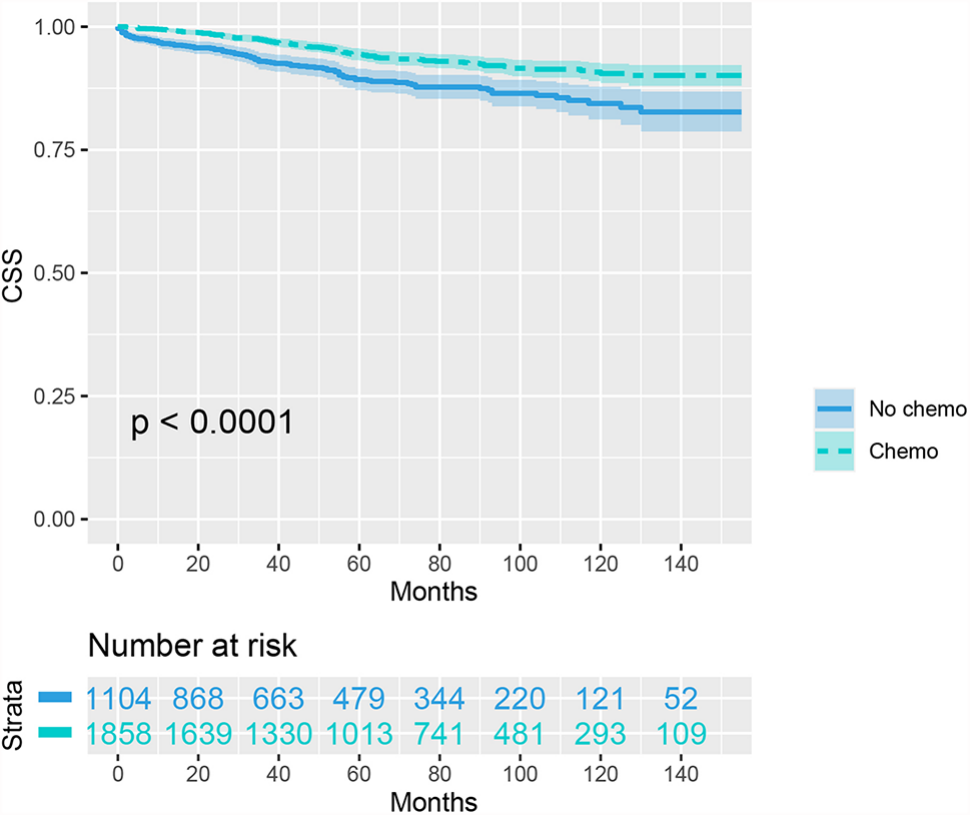
Kaplan-Meier plot, revealing cancer-specific survival differences based on the receipt of adjuvant chemotherapy in T1N+ colon cancer patients before propensity score matching.

We then included 2962 (8.1%) T1N+ colon cancer patients in further analyzes. As shown as **Figure 1**, the CSS curves of T1N+ colon cancer patients with and without the receipt of chemotherapy were generated using the Kaplan-Meier method. The CSS of T1N+ colon cancer patients with the receipt of chemotherapy was significantly better than those without the receipt of chemotherapy (94.3% VS. 89.3% for 5-year CSS rate, p < 0.001).

In an unadjusted Cox proportional hazards analysis, the cancer-specific mortality risk in patients with the receipt of chemotherapy was reduced by 48.1% (HR = 0.519, 95%CI =0.397-0.678, p < 0.001). Only variables with a P value less than 0.20 in unadjusted Cox analyses were then entered into multivariate Cox analyses, including information on the year of diagnosis, tumor location, age at diagnosis, patient race, gender, tumor grade, and whether they received chemotherapy. The results of multivariate analyses also showed that the cancer-specific mortality risk in patients with the receipt of chemotherapy was independently decreased by 46.0% (HR = 0.540, 95%CI =0.409-0.712, p < 0.001; **Table 2**).

**Table 2:** Cox regression analysis of prognostic factors for cancer-specific survival in T1N+ colon cancer.

Variables	Univariate analysis		Multivariate analysis	
	HR (95%CI)	*P value*	HR (95%CI)	*P value*
**Year of diagnosis**		0.097		0.179
** 2004**–**2007**	1		1	
** 2008**–**2011**	0.918 (0.682–1.236)	0.573	0.990 (0.735–1.335)	0.950
**2012**–**2015**	0.613 (0.393–0.957)	0.031	0.668 (0.427–1.046)	0.078
**Tumor location**		0.006		0.037
**Right-sided colon**	1		1	
** Left-sided colon**	0.686 (0.525–0.897)		0.744 (0.564–0.983)	
**Age at diagnosis (years)**		0.009		0.275
**≤65**	1		1	
**>65**	1.429 (1.093–1.869)		1.173 (0.881–1.561)	
**Number of lymph nodes harvested**		0.370		
**≤11**	1			
**≥12**	0.881 (0.669–1.161)			
**Race**		0.051		0.031
**White**	1		1	
** Black**	1.430 (1.004–2.036)	0.048	1.552 (1.086–2.218)	0.016
** Other**	0.741 (0.443–1.238)	0.252	0.825 (0.492–1.381)	0.464
**Gender**		0.127		0.102
** Male**	1		1	
** Female**	0.811 (0.620–1.061)		0.798 (0.609–1.045)	
**Grade**		0.044		0.035
** I/II**	1		1	
** III/IV**	1.490 (1.064–2.088)	0.020	1.482 (1.056–2.081)	0.023
**Unknown**	0.837 (0.475–1.473)	0.536	0.760 (0.431–1.340)	0.343
**Chemotherapy**		<0.001		<0.001
**No chemo**	1		1	
** Chemo**	0.519 (0.397–0.678)		0.540 (0.409–0.712)	

### The Efficacy of Chemotherapy in T1N+ Colon Cancer Patients After Propensity-Score Matching

As shown in **Table 3**, the clinicopathologic characteristics of T1N+ colon cancer patients were compared according to the receipt of chemotherapy before propensity-score matching. The year of diagnosis (35.2% VS. 29.3% for 2012-2015, p < 0.001), left-sided colon cancer (56.3% VS. 48.4% for left-sided colon, p < 0.001), younger age (62.5% VS. 34.8% for ≤ 65 years old, p < 0.001), more lymph nodes harvested (71.5% VS. 62.4% for ≥12 lymph node harvested, p < 0.001), and higher tumor grade (15.0% VS. 13.0% for grade III/IV, p < 0.001) were more prone to be associated with receipt of adjuvant chemotherapy in T1N+ colon cancer patients.

**Table 3:** Clinicopathologic characteristics of T1N+ colon cancer patients according to the receipt of chemotherapy before propensity score matching.

Variables	N0 chemo (N=1104)	Chemo (N = 1858)	*P value*
**Year of diagnosis**			<0.001
** 2004-2007**	411 (37.2%)	537 (28.9%)	
** 2008-2011**	370 (33.5%)	667 (35.9%)	
**2012-2015**	323 (29.3%)	654 (35.2%)	
**Tumor location**			<0.001
**Right-sided colon**	570 (51.6%)	812 (43.7%)	
** Left-sided colon**	534 (48.4%)	1046 (56.3%)	
**Age at diagnosis (years)**			<0.001
** ≤65**	384 (34.8%)	1,162 (62.5%)	
** >65**	720 (65.2%)	696 (37.5%)	
**Number of lymph nodes harvested**			<0.001
** ≤11**	415 (37.6%)	530 (28.5%)	
** ≥12**	689 (62.4%)	1,328 (71.5%)	
**Race**			0.090
**White**	864 (78.3%)	1,388 (74.7%)	
**Black**	137 (12.4%)	267 (14.4%)	
** Other**	103 (9.3%)	203 (10.9%)	
**Gender**			0.821
** Male**	552 (50.0%)	921 (49.6%)	
**Female**	552 (50.0%)	937 (50.4%)	
**Grade**			<0.001
**I/II**	849 (76.9%)	1,468 (79.0%)	
**III/IV**	143 (13.0%)	279 (15.0%)	
** Unknown**	112 (10.1%)	111 (6.0%)	

**Figure 2:**
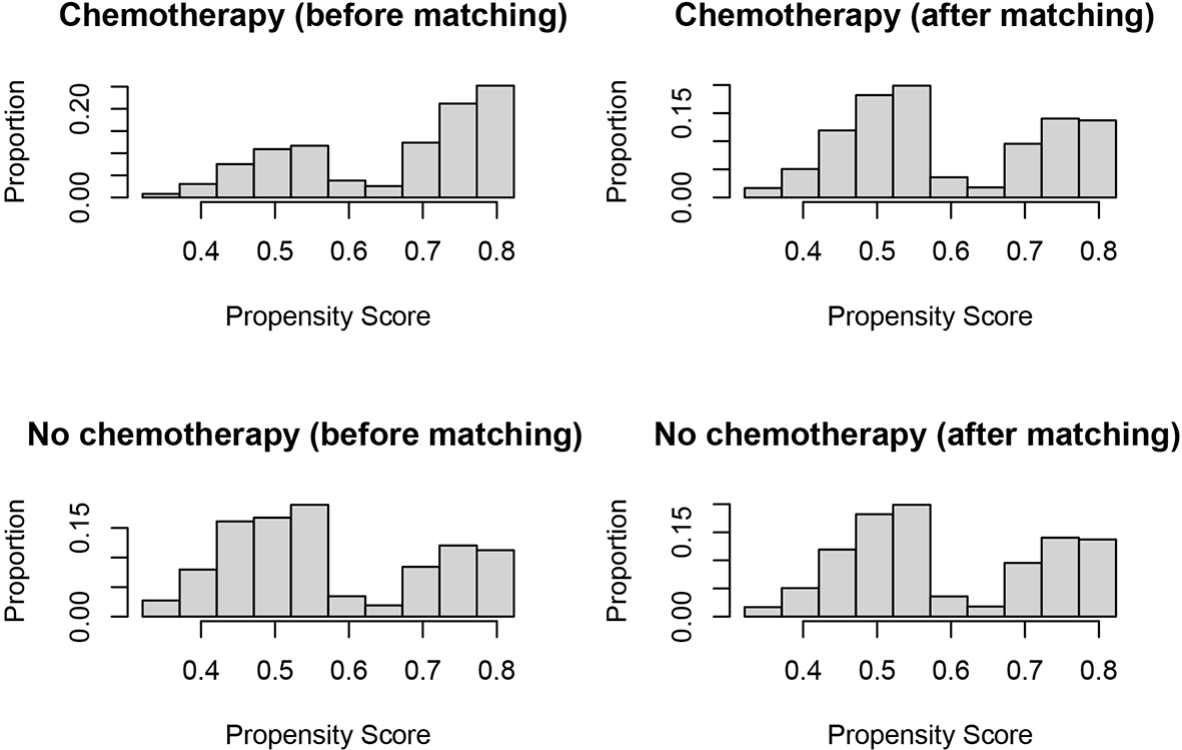
Distribution histograms before and after propensity score matching (treated = no surgery; control = radical surgery).

In evaluating the effect of chemotherapy on the survival of T1N+ colon cancer patients, to avoid the bias introduced by the retrospective design, we balanced the above demographic and clinical characteristics mentioned with propensity score matching. After matching by the ratio of 1:1, a total of 890 T1N+ colon cancer patients with the receipt of chemotherapy were matched to 890 T1N+ colon cancer patients without the receipt of chemotherapy. The distribution histograms before and after propensity-score matching are illustrated in **Figure 2**.

**Figure 3:**
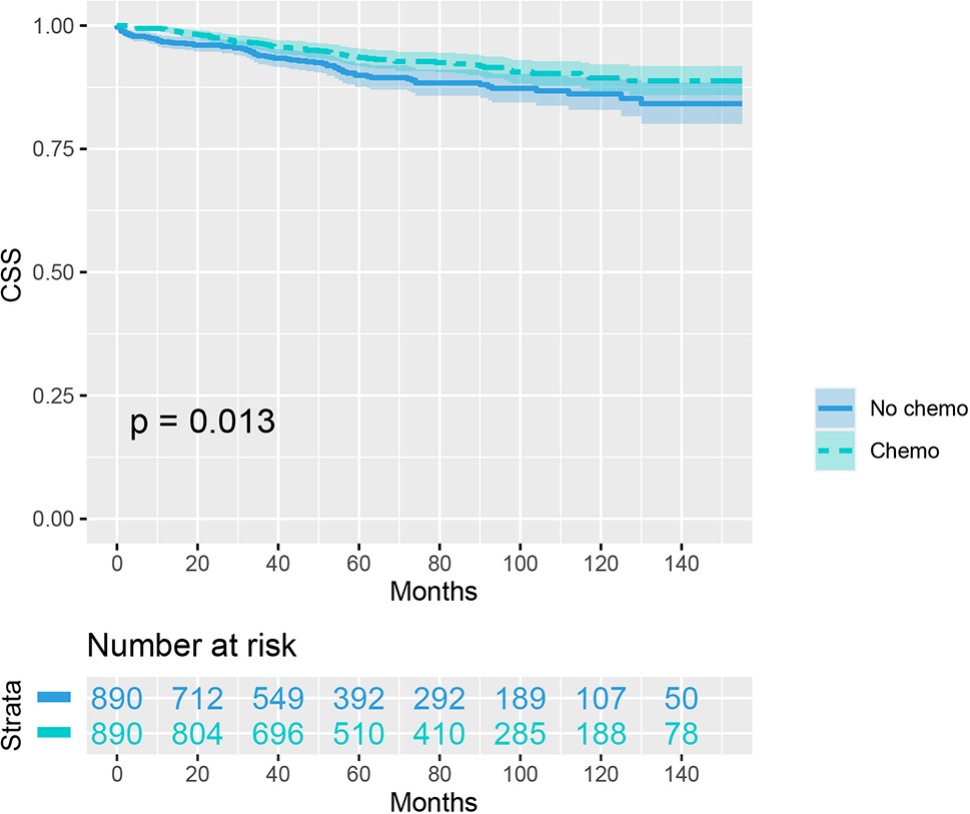
Kaplan-Meier plot, revealing the cancer-specific survival differences based on the receipt of adjuvant chemotherapy in T1N+ colon cancer patients after propensity score matching.

**Figure 4:**
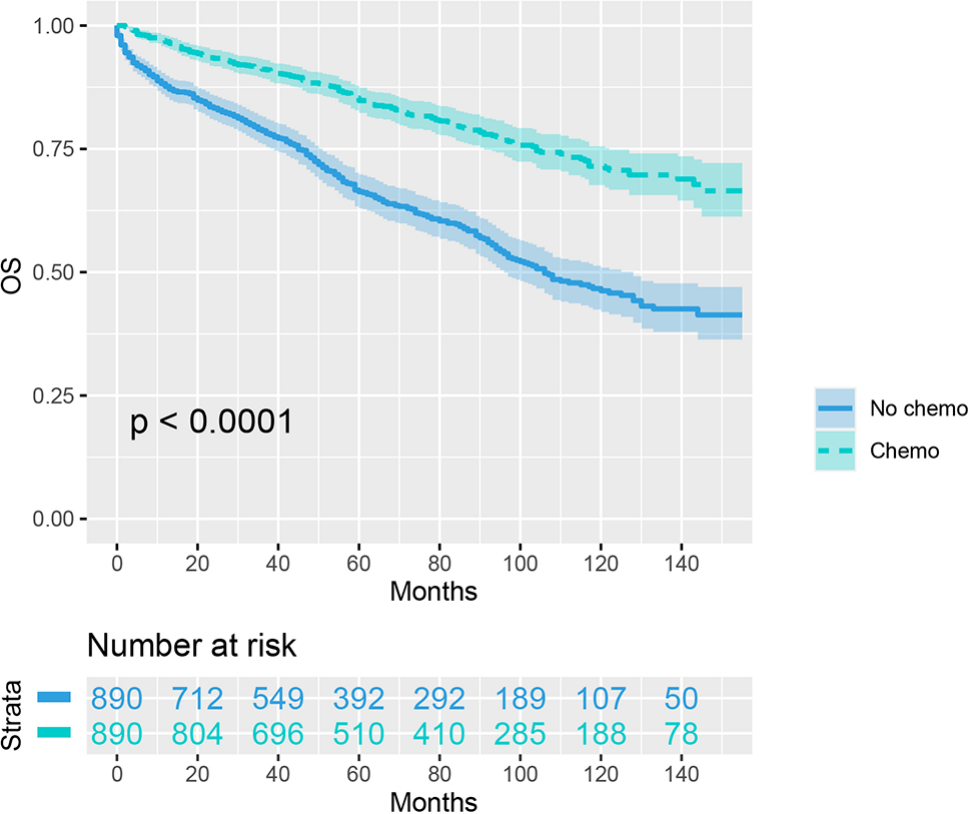
Kaplan-Meier plot, revealing the overall survival differences based on the receipt of adjuvant chemotherapy in T1N+ colon cancer patients after propensity score matching.

As indicated by **Table 4**, the clinicopathologic characteristics of T1N+ colon cancer patients were compared according to the receipt of chemotherapy after propensity-score matching. Our study found that there was no difference between both groups with regards to year of diagnosis (p = 1.000), tumor location (p = 1.000), age at diagnosis (p = 1.000), number of lymph nodes harvested (p = 1.000), patient race (p = 1.000), gender (p = 1.000) and tumor grade (p = 1.000). The receipt of adjuvant chemotherapy treatment following radical surgery did significantly decrease the risk of colon cancer-specific mortality by 33.9% after propensity-score matching (HR = 0.661, 95%CI = 0.476-0.917, p = 0.013). The Kaplan-Meier CSS curves of T1N+ colon cancer patients with and without the receipt of chemotherapy after propensity score matching are shown in **Figure 3**. The CSS of T1N+ colon cancer patients who received chemotherapy was significantly better than those who did not receive chemotherapy (93.5% VS. 89.9% for 5-year CSS rate, p = 0.013). Moreover, as seen in **Figure 4**, the OS of T1N+ colon cancer patients who received chemotherapy was significantly better than those who did not (84.8% VS. 66.3% for 5-year OS rate, p < 0.001).

**Table 4:** Clinicopathologic characteristics of T1N+ colon cancer patients according to the receipt of chemotherapy after propensity score matching.

Variables	N0 chemo (N = 890)	Chemo (N = 890)	*P value*
**Year of diagnosis**			1.000
** 2004-2007**	322 (36.2%)	322 (36.2%)	
** 2008-2011**	287 (32.2%)	287 (32.2%)	
** 2012-2015**	281 (31.6%)	281 (31.6%)	
**Tumor location**			1.000
**Right-sided colon**	440 (49.4%)	440 (49.4%)	
** Left-sided colon**	450 (50.6%)	450 (50.6%)	
**Age at diagnosis (years)**			1.000
**≤65**	356 (40.0%)	356 (40.0%)	
**>65**	534 (60.0%)	534 (60.0%)	
**Number of lymph nodes harvested**			1.000
**≤11**	282 (31.7%)	282 (31.7%)	
** ≥12**	608 (68.3%)	608 (68.3%)	
**Race**			1.000
**White**	715 (80.3%)	715 (80.3%)	
**Black**	106 (11.9%)	106 (11.9%)	
** Other**	69 (7.8%)	69 (7.8%)	
**Gender**			1.000
** Male**	445 (50.0%)	445 (50.0%)	
** Female**	445 (50.0%)	445 (50.0%)	
**Grade**			1.000
**I/II**	717 (80.6%)	717 (80.6%)	
**III/IV**	121 (13.6%)	121 (13.6%)	
** Unknown**	52 (5.8%)	52 (5.8%)	

## Discussion

In colon cancer, the presence of lymph node metastasis is a prognostic feature in poor prognosis configuration. In theory, lymph node metastasis should not occur when the tumor is confined to the mucosal layer because this layer is devoid of lymphatic vessels. T1 colon cancer, which refers to carcinoma with invasion confined to the submucosa, however, had an approximately 10% probability of experiencing lymph node metastases and therefore requires radical intestinal resection with lymph node dissection. Many studies have previously evaluated the risk factors for lymph node metastasis in T1 colon cancer, however, differences in opinion have always existed (3, 5, 17–21).

In our analyses, younger age, female gender, more lymph nodes harvested, Black race and higher tumor grade were more prone to be diagnosed with lymph node involvement. Two recent studies have reported that young age at diagnosis could be associated with an increased risk of lymph node involvement and more aggressive screening and postoperative treatments should be considered for young patients with T1 colon adenocarcinoma/ (22, 23) This phenomenon might be due to the potential genetic differences between young and elderly patients, as young patients are more likely to present with more aggressive features and adverse histological grades (24–26) In line with the results of previous studies, we found that Black race had a risk factor of developing metastasis (23). The higher rate of lymph node metastasis in female colon cancer patients diagnosed with T1 disease might result from the sex hormones between male and female patients (27, 28). It has also been observed that T1 carcinoma located in the left-sided colon shows higher rates of lymph node metastasis than right-sided colon, though it was not statistically significant in our study (29,30).

According to current clinical guidelines, T1 colon cancer patients with lymph node metastasis should receive adjuvant chemotherapy following radical surgery. Moreover, 5‐FU‐based chemotherapy has been demonstrated to have significant survival benefits for patients with lymph node metastasis (31–33). Early in 1990, Moertel and collaborators (8) demonstrated an improved prognosis of chemotherapy in colon carcinoma with lymph node metastasis following radical resection. Later in 2004, the famous MOSAIC study proposed that adding oxaliplatin to a regimen of fluorouracil and leucovorin provides improved efficacy in the adjuvant treatment of colon cancer (9). Cases of T1 disease are relatively few and account for a small proportion of colon cancer. It has been reported that such patients account for 2 to 12 percent of all colon cancer patients in colonoscopic studies (11–15). It is important to note that the above studies evaluating the efficacy of adjuvant chemotherapy for stage III colon cancer did not include T1 disease. However, T1 colon cancer patients with lymph node involvement following radical resection often did not receive further chemotherapy after surgery and the available data of oncological outcomes in T1 node-positive (N+) patients is lacking (10). In 2005, Wang et al. (34) evaluated the prognosis of T1 colorectal cancer in a small population (n = 159) and found that predictive factors for the risk of lymph node metastasis in T1 colorectal cancer after radical resection do not impact the long-term prognosis.

Despite findings such as these, studies on T1 colon cancer patients are mostly focused on the predictive factors for the risk of lymph node metastasis following radical resection (5, 6). T1 disease with lymph node involvement is much rarer than without lymph node metastasis, therefore, a large cancer database was required to examine the efficacy of chemotherapy in such patients. More importantly, our study has shown that adjuvant chemotherapy treatment could provide significantly better oncological outcomes in T1 colon cancer patients with lymph node involvement following radical surgery. In an unadjusted Cox proportional hazards analysis, the cancer-specific mortality risk in patients with the receipt of chemotherapy was reduced by 48.1% (p < 0.001). The results of multivariate analyses also showed that the cancer-specific mortality risk in patients who received chemotherapy was independently reduced by 46.0% (p < 0.001). In addition, to reduce the bias introduced by the retrospective design, we balanced the demographic and clinical characteristics with propensity-score matching. The receipt of adjuvant chemotherapy treatment following radical surgery significantly reduced the risk of colon cancer-specific mortality by 33.9%, even after propensity-score matching. Kaplan-Meier analysis also showed that the CSS of T1N+ colon cancer patients with the receipt of chemotherapy was significantly better than those without the receipt of chemotherapy after propensity score matching, and the 5-year CSS rates were 93.5%, and 89.9%, respectively (p = 0.013).

The major strengths of the current study are that it used a large cohort, and that we validated that younger age, female gender, more lymph nodes harvested, Black race, and higher tumor grade are more prone to be diagnosed with lymph node involvement. More importantly, by employing propensity score matching, the study provides a high level of evidence that the receipt of adjuvant chemotherapy following radical surgery significantly decreases the risk of colon cancer specific mortality by 33.9% in T1 colon cancer with lymph node involvement.

Several limitations of the current study should also be noted. First, the information on postoperative complications, which could negatively affect the prognosis of colon cancer patients after radical resection was not included in the database, and could cause potential systematic bias. Second, the drawbacks introduced by the retrospective design could not be avoided, even though propensity-score matching was used. Finally, this database did not provide information on specific chemotherapy regimens, and further large-scale studies evaluating the effect of different chemotherapy regimens on survival in T1 colon cancer patients with lymph node metastasis are required.

## Conclusions

Patients of younger age, female gender, more lymph nodes harvested, Black race, and higher tumor grade are more prone to be diagnosed with lymph node involvement. Using propensity-score matching, this study has provided important evidence that the receipt of adjuvant chemotherapy following radical surgery could significantly decrease the risk of colon cancer-specific mortality by 33.9% in T1 colon cancer with lymph node involvement.

## Data Availability Statement

Publicly available datasets were analyzed in this study. This data can be found here: https://seer.cancer.gov/.

## Author Contributions

WY and QL conceived the project and wrote the manuscript. SS and JL collected the data. WY, HZ, and SS undertook the analysis. All authors contributed to the article and approved the submitted version.

## Conflict of Interest

The authors declare that the research was conducted in the absence of any commercial or financial relationships that could be construed as a potential conflict of interest.

## Publisher’s Note

All claims expressed in this article are solely those of the authors and do not necessarily represent those of their affiliated organizations, or those of the publisher, the editors and the reviewers. Any product that may be evaluated in this article, or claim that may be made by its manufacturer, is not guaranteed or endorsed by the publisher.
