# Supplementary figures and images for: Association Between Chemotherapy and Survival in T1 Colon Cancer With Lymph Node Metastasis: A Propensity-Score Matched Analysis
Source: Front Oncol. 2021 Jul 30;11:699400. doi: 10.3389/fonc.2021.699400 (PMC8361445; doi:10.3389/fonc.2021.699400)

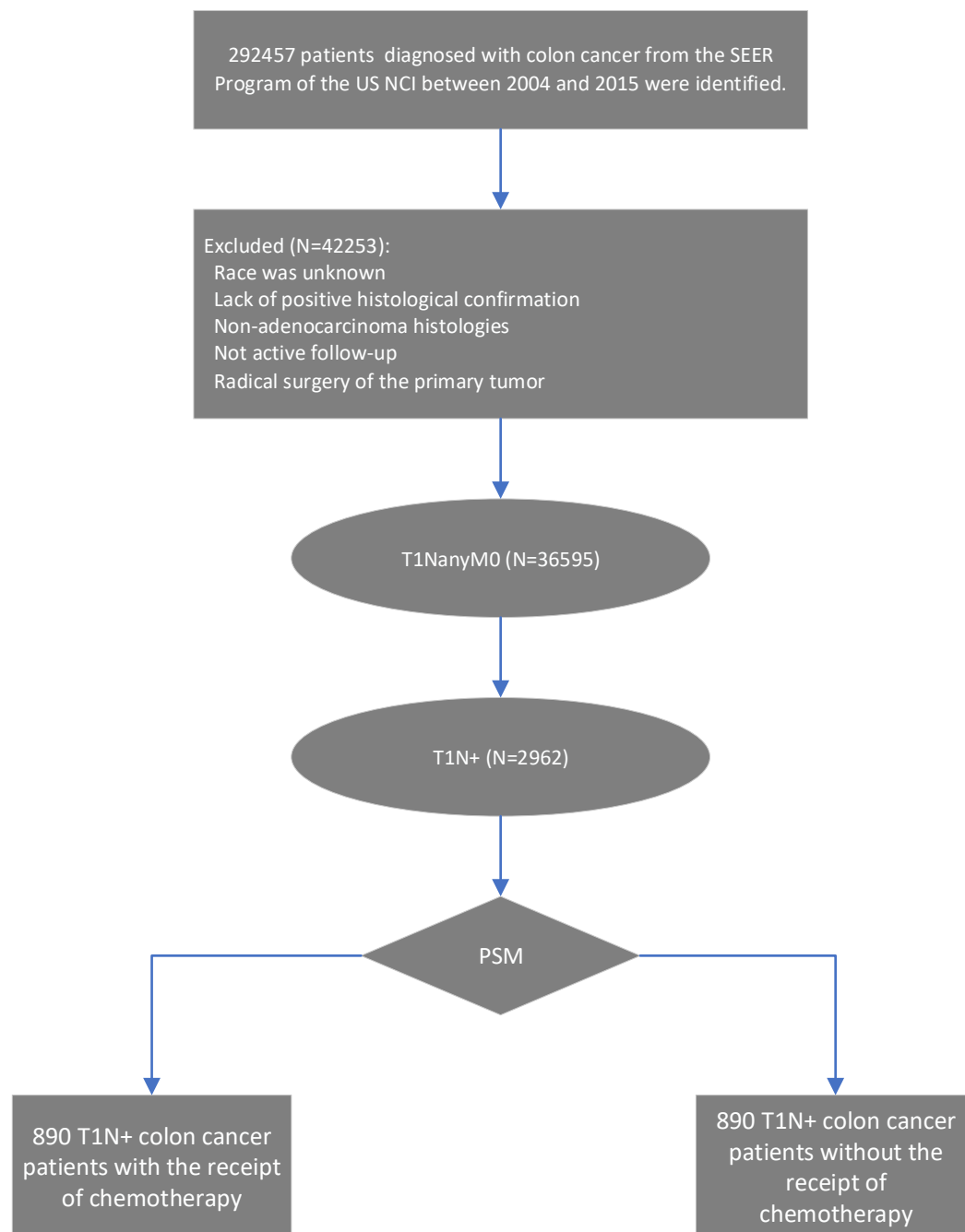

Supplement: Supplementary file 1 [file DataSheet_1.pdf]
